# A novel survival model of cardioplegic arrest and cardiopulmonary bypass in rats: a methodology paper

**DOI:** 10.1186/1749-8090-3-51

**Published:** 2008-08-19

**Authors:** Fellery de Lange, Kenji Yoshitani, Mihai V Podgoreanu, Hilary P Grocott, G Burkhard Mackensen

**Affiliations:** 1 Department of Anesthesiology, Duke University Medical Center, Durham, North Carolina, USA; 2 Division of Perioperative Care and Emergency Medicine, University Medical Center Utrecht, Utrecht, The Netherlands; 3 Department of Anesthesia, University of Manitoba, Winnipeg, Manitoba, Canada

## Abstract

**Background:**

Given the growing population of cardiac surgery patients with impaired preoperative cardiac function and rapidly expanding surgical techniques, continued efforts to improve myocardial protection strategies are warranted. Prior research is mostly limited to either large animal models or *ex vivo *preparations. We developed a new *in vivo *survival model that combines administration of antegrade cardioplegia with endoaortic crossclamping during cardiopulmonary bypass (CPB) in the rat.

**Methods:**

Sprague-Dawley rats were cannulated for CPB (n = 10). With ultrasound guidance, a 3.5 mm balloon angioplasty catheter was positioned via the right common carotid artery with its tip proximal to the aortic valve. To initiate cardioplegic arrest, the balloon was inflated and cardioplegia solution injected. After 30 min of cardioplegic arrest, the balloon was deflated, ventilation resumed, and rats were weaned from CPB and recovered. To rule out any evidence of cerebral ischemia due to right carotid artery ligation, animals were neurologically tested on postoperative day 14, and their brains histologically assessed.

**Results:**

Thirty minutes of cardioplegic arrest was successfully established in all animals. Functional assessment revealed no neurologic deficits, and histology demonstrated no gross neuronal damage.

**Conclusion:**

This novel small animal CPB model with cardioplegic arrest allows for both the study of myocardial ischemia-reperfusion injury as well as new cardioprotective strategies. Major advantages of this model include its overall feasibility and cost effectiveness. In future experiments long-term echocardiographic outcomes as well as enzymatic, genetic, and histologic characterization of myocardial injury can be assessed. In the field of myocardial protection, rodent models will be an important avenue of research.

## Background

Although considerable progress has been made in surgical techniques and other perioperative management to allow for the majority of patients to undergo cardiac surgery without significant mortality, more than 25% of this surgical population may still experience substantial morbidity related to adverse cardiovascular events. These include prolonged contractile dysfunction (stunning), myocardial infarction, low-output syndromes, and overt ventricular failure, all resulting in prolonged intensive care unit stay and reduced functional capacity at discharge, and ultimately contribute to overall mortality [1,2]. Mortality after perioperative myocardial infarction is 40–50% [3]. The etiology of myocardial dysfunction following cardiac surgery is multifactorial but frequently involves perioperative myocardial ischemia reperfusion injury [4].

Since the advent of cardiopulmonary bypass (CPB), cardioplegic arrest has been an essential component of cardiac surgery but remains associated with ischemia-reperfusion injury to the myocardium [5-7]. As the population ages and percutaneous coronary interventions have become a standard therapy, patients referred for cardiac surgery generally present with a higher risk for perioperative cardiovascular complications [8,9]. These complications are primarily due to increased comorbidities and more complex surgical interventions resulting in the need for more prolonged aortic crossclamp and CPB times, all making optimized myocardial protection strategies an essential component of cardiac surgery procedures. Experimental efforts to better understand the underlying mechanism associated with postoperative myocardial reperfusion injury and to improve established myocardial protection protocols have been limited to either costly large animal models or *ex vivo *heart preparations [10-12]. The use of normothermic cardioplegia solutions has shown beneficial results, but research is limited and principally relies on isolated heart models [13-15]. However, these models do not facilitate research on long-term effects of myocardial reperfusion injury or novel therapeutic interventions. To advance the field, additional research in a suitable rodent model with good survivability appears to be warranted. Such a model would not only allow to further elucidate mechanisms of adverse myocardial outcomes following cardioplegic arrest but also permits the characterization of genetic, proteomic, and histologic changes as well as long-term functional outcomes in response to injury and therapy. It might also facilitate further research aiming to optimize current cardioprotective strategies *in vivo *and facilitate myocardial gene delivery studies [16,17].

Based on an existing beating-heart model of CPB in the rat [18], we developed a novel *in vivo *survival model that allows administration of antegrade cardioplegia and endoaortic crossclamping. To rule out any gross neurological damage due to cannulation of the right carotid artery, a functional assessment and histological evaluation of the brains was performed.

## Methods

The study was approved by the Duke University Animal Care and Use Committee, and all procedures met the National Institutes of Health (NIH) guidelines for animal care [19].

Male 400–425 g Sprague-Dawley rats (Charles River Labs, Wilmington, MA, USA) were housed two per cage under a 12-hour light-dark cycle with food and water available ad libitum.

### Anesthesia, surgical preparation, cardiopulmonary bypass, and neurological assessment

Fasted rats (n = 10) were anesthetized with 5% isoflurane in 50% O_2 _in a plastic induction box. After orotracheal intubation with a 14G cannula (Insyte BD Medical, Sandy, UT), the animals were mechanically ventilated (Harvard Model 687, Harvard Apparatus, Holliston, MA) 60 breaths·min^-1 ^with FiO_2 _0.6 while maintaining a normal arterial carbon dioxide tension. The maximal airway pressure did not exceed 20 mmH_2_O. During subsequent surgical preparation, anesthesia was maintained with 1.5–2.0% isoflurane. A needle thermistor was inserted in the left temporal muscle adjacent to the skull to measure pericranial temperature. With both forced-air and surface heating systems, temperature during surgery was controlled at 36°C when not on CPB, during CPB, and cardioplegic arrest at 34°C. Towards the end of, and after CPB, temperature was controlled at 37°C. Electrocardiogram (EKG) electrodes were placed on both front paws and left hind paw.

**Figure 1:**
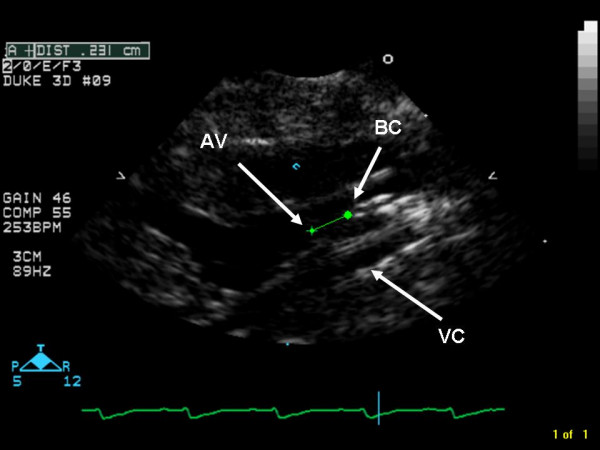
**Echocardiographic image of angioplasty balloon catheter positioned near aortic valve.** Green distance measure indicating a distance of 0.23 cm from tip of balloon catheter to aortic valve. AV = aortic valve, BC = tip of balloon catheter, VC = multi-orificed venous cannula advanced into right atrium.

Surgical preparation consisted of cannulation of the tail artery with a 20 G catheter (Insyte BD Medical, Sandy, UT), which served as the arterial inflow cannula for the CPB circuit. 150 IU porcine heparin and 5 μg fentanyl were administered after placement of this first cannula. Mean arterial blood pressure was monitored via the superficial caudal epigastric artery, which was cannulated with polyethylene tubing (PE-10 Intramedic Tubing, Becton-Dickinson, Sparks, MD). A 3.5 mm angioplasty balloon catheter (Sprinter^® ^OTW, Medtronic Inc, Minneapolis, MN) was retrogradely inserted into the ascending aorta via the right common carotid artery and positioned, under ultrasound guidance (SONOS 7500, Philips Medical Systems, Andover, MA), with its tip just above the aortic valve (Figure 1). This catheter served as an endoaortic crossclamp during the experiment, comparable with PortAccess™ surgery as performed in humans [20]. A multi-orificed 4.5 Fr catheter (modified Desilets-Hoffman Catheter, Cook, Bloomington, IN) was advanced through the right external jugular vein into the right atrium and served as a conduit for venous outflow. Repeat injections of 150 IU porcine heparin and 5 μg fentanyl were administered, and CPB was initiated. In addition, 0.2 mg pancuronium was administered prior to CPB. Muscle relaxation was used to prevent spontaneous ventilation that often interferes with venous return due to movement of the mediastinal structures relative to the venous outflow cannula.

**Figure 2:**
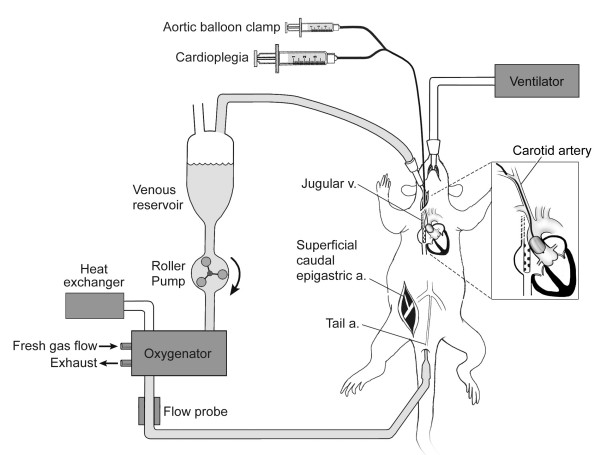
**Schematic diagram of rat CPB apparatus and surgical preparation highlighting the aortic balloon catheter serving as endoaortic crossclamp.** CPB = cardiopulmonary bypass.

The CPB circuit (Figure 2) consisted of a 4 ml Plexiglas venous reservoir, a roller pump (Masterflex; Cole-Parmer Instrument Co., Vernon Hills, IL), and a custom-designed small-volume oxygenator (M. Humbs, Valley, Germany). The 4 ml priming volume oxygenator was built of two Plexiglas shells (12.8 cm × 12.8 cm × 2.7 cm) that carry a sterile, disposable three layer hollow fiber membrane providing a surface area for gas exchange of 558 cm^2 ^[18,21]. To prevent excessive heat loss, one of the shells had an integrated heat exchanger. An in-line flow probe (2N806 probe and T208 flowmeter, Transonics Systems Inc., Ithaca, NY) was used to continuously measure CPB flow. The entire circuit was primed with 10 mL of 6% hetastarch (Hextend, Hospira Inc, Lake Forest, IL). All parts were connected through single use silicone tubing. During CPB, a flow rate of 150 mL·kg^-1^·min^-1 ^was maintained, and an average of 3 mL of hetastach was added to compensate losses and extravasation. At the start of CPB, frequency of ventilation was lowered to 30 min^-1^, and 0.5–1% isoflurane was administered through the oxygenator with 70% oxygen and additional CO_2 _as needed (α stat blood gas management). After 15 min of CPB, the endoaortic clamp was quickly inflated while concomitantly 0.5 ml cardioplegia was infused via an infusion pump at a rate of 200 mL·hr^-1 ^through the central lumen of the angioplasty catheter. Cardioplegia solution consisted of 4 parts of standard undiluted adult cardioplegia induction solution (5% dextrose in 0.225% NaCl (655 mL·L^-1^) potassium chloride (95 mEq·L^-1^), tromethamine (238 mL·L^-1^), and citrate-phosphate-dextrose (CPD) solutions (60 mL·L^-1^), and 1 part esmolol 10 mg·ml^-1^. Ventilation was discontinued. Cardioplegic arrest was confirmed both by ultrasound and electrocardiographically. The CPB flow rate was adjusted as needed to maintain a constant venous reservoir blood level. At 15 min of arrest, a second dose of 0.4 mL of cardioplegia was administered to uphold the arrest. After 30 min of cardioplegic arrest, the balloon was deflated and removed and ventilation restarted at a slower rate of 35 min^-1 ^and FiO_2 _of 0.7. CPB was maintained for another 30 min to allow for rewarming of the animals. After discontinuation of CPB, the ventilatory rate was raised to 60 min^-1^. The venous cannula was removed, and the incision closed. During the entire experiment, mean arterial pressure was maintained above 45 mmHg, with the use of small doses of phenylephrine if necessary. After cessation of CPB, the remaining blood left in the CPB circuit was centrifuged for 5 min at 3000 rpm, and the supernatant discarded. Two mL of the remaining red blood cell concentrate was then re-infused. The animals were ventilated for another 60 min, after which the remaining cannulae were removed, and the wounds closed. The rats recovered in a warmed and oxygen-enriched environment for at least 12 hr prior to return to their home cages. Blood gas analysis was performed before the start of CPB, at 15 min of CPB, at 15 min of arrest, at the end of 30 min arrest, at the end of the total of 75 min of CPB, and at 1 hr post CPB, using an IL-GEM Premier 3000 blood gas analyzer (Global Medical Instrumentation, Ramsey, MI).

During the first 14 postoperative days, the animals were checked daily for their overall well-being and wound healing. To determine if any neurologic injury occurred (because of intraoperative usage and postoperative ligation of the right common carotid artery), neurological function was assessed at day 14, using a previously established neurological scoring system [22]. In brief, it was derived by evaluating four different functions (general status, simple motor deficit, complex motor deficit, and sensory deficit). The score given to each animal was the sum of all four individual scores: 0 was the best and 48 the worst score possible [22].

### TTC staining

On postoperative day 14, the animals were sacrificed by inhalation of 5% isoflurane followed by decapitation. The brains were serially sliced into 2 mm coronal sections with the use of a brain matrix. Brain sections were immediately incubated in TTC (2,3,5-Triphenyl-tetra-zolium-chlorid) at 37°C for 20 minutes and subsequently stored in 10% phosphate-buffered formalin to detect any focal cerebral injury.

## Results

Full cardioplegic arrest over 30 min was successfully achieved in all animals. In two out of the ten animals, the initial arrest after the induction bolus of cardioplegia was incomplete as seen on the EKG. However, with a subsequent dose of 0.2 mL of cardioplegia, full arrest was achieved. None of the animals received more than 0.9 mL of cardioplegia in total. Following 30 min of cardioplegic arrest, when the endoaortic balloon was deflated, spontaneous cardiac rhythm with frequencies identical as pre-CPB resumed within seconds. Separation from CPB without the use of inotropic agents was achieved in all animals.

Table 1 displays the physiologic parameters of all animals. Because of the nature of CPB, rats demonstrated lower MAP, higher PaO_2 _values, and lower hemoglobin concentrations during CPB as compared to baseline values. Retransfusion of 2 ml of concentrated blood from the CPB circuitry allowed the hemoglobin level to rise again. All animals survived the postoperative period and underwent neurological testing at day 14. Out of the 48 point maximum (worst) score, the animals scored 2.5 ± 1.4 points on average, consistent with no neurological deficits. Histological assessment of the brain showed no infarcted areas in any of the animals.

**Table 1:** Physiologic parameters.

		**CPB**				
			
	**Baseline**	**15 CPB**	**15 arrest**	**30 arrest**	**75 CPB**	**1 h post**
**Weight (g)**	419 (7.7)					
**Hematocrit (%)**	41 (1)	27 (2)	28 (2.7)	29 (2.1)	29 (3)	33 (2)
**Glucose (mg/dl)**	100 (14)				136 (12)	
**Temperature (°C)**	35.6 (0.6)	34.8 (0.6)	34.4 (0.5)	34.2 (0.4)	35.6 (0.7)	36.9 (0.5)
**CPB flow (ml/min)**		51 (5)	65 (9)	62 (11)		
**MAP (mmHg)**	62 (7)	61 (5)	48 (5)	46 (5)	68 (15)	75 (8)
**Arterial pH**	7.42 (0.02)	7.42 (0.06)	7.46 (0.06)	7.43 (0.08)	7.38 (0.08)	7.38 (0.06)
**PaC0_2 _(mmHg)**	40 (2)	35 (5)	34 (5)	36 (5)	42 (7)	41 (5)
**Pa0_2 _(mmHg)**	208 (33)	366 (52)	348 (69)	325 (107)	308 (68)	285 (63)
**HCO_3_^- ^(mmHg)**	26.1 (1.6)	22.5 (1.4)	23.9 (2.1)	24.2 (1.8)	25.6 (2.0)	24.4 (3.0)

CPB = cardiopulmonary bypass

MAP = mean arterial pressure

PaC0_2 _= Partial pressure of carbon dioxide

Pa0_2 _= Partial pressure of oxygen

HC0_3 _= Standard bicarbonate

## Discussion

Research focusing on cardioprotective strategies during cardiac surgery has been hindered by the lack of a suitable small animal model that would allow for complete cardioplegic arrest with good survivability. Most previous research was performed in isolated heart models [10,14,15]. While these models allow investigating the immediate effects of therapeutic interventions or different cardioplegia solutions, they preclude the assessment of long-term histological, biochemical, or functional outcomes. Survival studies using dogs [13,23,24] or pigs [25] have been performed but are limited due to sample size and costs. Although a number of rat CPB models have been described over the years [26-30], all of them were resembling beating heart CPB, and none of them included any form of aortic crossclamping and antegrade cardioplegia administration. To our knowledge, only one small animal cardioplegic arrest model has been described so far [31]. In their paper, Günzinger et al. describe a rat model in which cardioplegic arrest is achieved by injecting a cardioplegic solution into the aortic through a sternotomy while at the same time the branchiocephalic trunk and aortic arch are occluded by tourniquets. Blood loss during the procedure is significant. Compared to our model, this previously described model is not a survival model as it includes full sternotomy (preferably avoided in small four-legged animals), and carotid arteries and jugular veins are cannulated. Therefore, we describe a novel *in vivo *survival CPB model that allows minimal invasive administration of antegrade cardioplegia with endoaortic crossclamping with resulting cardioplegic arrest in rats.

Due to the excellent survivability and ease of postoperative cardiac recovery, this model lends itself to the investigation of genomic and proteomic changes as well as histological alterations that can be assessed at any time point and new therapeutic interventions aiming to optimize cardioprotection. Over the last several years, substantial preclinical advances have been made in gene- or cell-based therapies for myocardial protection and in rescue strategies for myocardial ischemia-reperfusion injury, all aimed to employ different types of genes, vectors, and delivery routes [32,33]. Among these, cardiac gene delivery methods with the use of CPB, in which the adenoviral vector is administered following cardioplegic arrest, allow prolonged myocardial exposure time to the adenoviral vector and improved gene transfer [16,17]. We speculate that the model described here will, therefore, facilitate direct intracoronary administration of medications, gene vectors, or cells and might even allow for ultrasound-mediated gene transfer. Because exposure time and coronary flow are major determinants of efficient intracoronary delivery, complete cardioplegic arrest with negligible coronary flow and long wash-in periods will likely optimize delivery and limit extracardiac expression [16,17].

In the search for a suitable model to study novel cardioprotective strategies, we adapted a rodent CPB model that we had previously described and utilized [34,35]. In the original description by Grocott et al.,[29] the smallest available human oxygenator was used, which was largely oversized for rodents. The model was later adapted, and a small, appropriately sized rat oxygenator was developed and inserted, thereby abolishing the need of donor blood to prime the circuit and allowing effects of appropriately sized CPB to be determined [18,36]. Careful positioning of the venous outflow catheter allowed for optimal venous drainage, and CPB flows consistent with a normal cardiac output in rats could be achieved. The natural cardiac rhythm, however, was unaltered, and pilot studies indicated that when venous drainage was not optimal, the heart continued to variably eject. Therefore, we adapted this model by inserting the smallest commercially available angioplasty balloon catheter with a central lumen retrogradely into the common carotid artery with the tip carefully positioned just proximal to the aortic valve. Inflation of the balloon resulted in effective aortic crossclamping, and concomitant administration of cardioplegia solutions through the lumen allowed for immediate cardioplegic arrest confirmed by ultrasound and EKG.

The right common carotid artery provided direct access to the ascending aorta, combining the correct vessel size with a relatively short distance to the aortic valve. A full sternotomy and direct cannulation of the heart is very invasive and would likely cause increased mortality. At the end of the experiment when the catheter is removed, the right common carotid artery is permanently ligated. Although it has been described that ligation of one carotid artery in young healthy rats is without consequences [37], the exclusion of gross neurological or histological abnormalities appeared to be justified before utilizing or developing this model further. The absence of neurological deficits screened with a 48-point neurologic scoring system [22], and the absence of any signs of cerebral infarction following TTC staining of the brains 14 days after surgery support this prior work. Because the scope of the current work was purely to develop the experimental technique and demonstrate the technical feasibility of achieving 30 min of full cardioplegic arrest in rats with good survivability, we did not quantify cardiac function postoperatively. However, the model described here permits comprehensive perioperative echocardiographic evaluation of cardiac function in future studies. Another major advantage of this model is its minimal invasiveness (closed chest), and that it can be performed by one operator only, thus facilitating higher experimental throughput at lower costs. As an indication, the disposable membrane for the oxygenator costs $65, the Plexiglas parts of the CPB system (venous reservoir, oxygenator shell) can be re-used after vigorous cleaning and sterilizing while the CPB tubing was discarded after each bypass run. After extensive training, a perioperative mortality less than 10–15% can be achieved.

The model has several potential pitfalls. The endoaortic catheter requires exact and precise positioning close to the aortic valve. Ultrasonographic imaging is essential for positioning of the angioplasty catheter as well as assessing the cardioplegic arrest. When the catheter is not positioned optimally, incomplete arrest will be the result, and the balloon might even damage the brachiocephalic trunk (too high) or the left ventricle (too deep). If the injection of cardioplegia is executed too forcefully, dilatation of the left ventricle will occur. Measuring the pressure at the tip of the balloon catheter combined with the use of an infusion pump solves this issue. Use of the manufacturer's inflation device prevents overinflation of the endoaortic balloon. After failed cardioplegic arrest on the first attempt, repeated small doses of cardioplegia will result in complete arrest. Although we did not measure potassium in these animals, earlier pilot experiments revealed that hyperkalemia does not occur with the dosing regimen used in this protocol. Immediate return to regular sinus rhythm at preoperative rates following deflation of the endoaortic balloon and restoration of preoperative hemodynamics present further indirect evidence that the cardioplegic regimen did not result in hyperkalemia.

As this work was entirely focused on developing the minimal invasive technique to accomplish CPB with cardioplegic arrest, we have not yet systematically investigated possible definite outcomes. However, study endpoints such as technical feasibility, effective cardioplegia, and survival were successfully demonstrated. The model described will not only facilitate further research to elucidate mechanisms of myocardial reperfusion injury following cardioplegic arrest and CPB but also to evaluate novel approaches to improved myocardial protection. Due to its minimal invasiveness and ease of recoverability, short- and long-term effects of constituents of cardioplegia, duration of cardioplegic arrest, CPB time, and potentially also direct gene transfer on myocardial function and histological outcomes can be assessed better than in isolated heart models. In addition, the model allows for the investigation of unique animal strains with varying susceptibility to myocardial injury depending on either their genetic background (consomic) or preexisting disease (e.g., diabetes, old age, hypertension).

## Conclusion

This novel small animal CPB model with cardioplegic arrest allows for both the study of myocardial ischemia-reperfusion injury as well as the evaluation of new cardioprotective strategies. Major advantages of this model include its overall feasibility and cost effectiveness. In the field of myocardial protection, rodent models will remain an important avenue of research. Models such as the one described here will likely be utilized to not only assess longer-term functional outcomes but also characterize the enzymatic, genetic, and histologic response to myocardial injury and protective strategies.

## Competing interests

The authors declare that they have no competing interests.

## Authors' contributions

FD performed animal surgery, was involved in development of the model, analysis, and interpretation of the work. Major role in preparation of the manuscript. KY participated in execution of the experiments and histology. MP contributed to development of the model and also contributed to the manuscript. HG contributed to design and interpretation of the studies, also contributed to the manuscript. GM participated in design, development of the model, execution, analysis, and interpretation of the work. Major role in preparation of the manuscript. All authors read and approved the final manuscript.
